# How Our Caregivers Shape Who We Are: The Seven Dimensions of Attachment at the Core of Personality

**DOI:** 10.3389/fpsyg.2021.657628

**Published:** 2021-07-01

**Authors:** Marcantonio Gagliardi

**Affiliations:** Department of Computer Science, The University of Sheffield, Sheffield, United Kingdom

**Keywords:** attachment, personality, psychopathology, cognitive, representation, dimensions, core beliefs, imprinting

## Abstract

Psychology defines personality as the stable traits of an individual, and cognitive research suggests that a set of core beliefs is at the root of these traits. From this perspective, two major questions remain unanswered: (1) What are the core beliefs that make up personality? (2) How are they acquired? An interesting answer is provided by attachment theory, according to which attachment is at the basis of personality. The current theoretical formulation, however, does not sufficiently clarify the relationship between the two. Adopting a cognitive-clinical approach, we put forward a novel version of attachment theory, arguing that it can better account for the relationship between attachment and personality, thereby providing more convincing answers to questions (1) and (2). In particular, we propose that: (A) attachment information is acquired over seven dimensions; (B) the acquisition of each dimension is induced by a specific caregiving feature and (C) realized through a specific acquisition mechanism – imprinting. In a nutshell, we propose an Attachment-Personality Model (APM) according to which seven attachment dimensions constitute the knowledge core of personality. We finally discuss the significant implications of the model, especially its clinical application in terms of conception, assessment, and treatment of mental disorders. The model can be empirically tested, and we suggest three ways to do that.

## Introduction

Psychologists usually recognize the significant explanatory power of attachment theory (Cassidy and Shaver, 1999, 2008, 2016) and the clinical effectiveness of cognitive psychotherapy (Beck, 2011; Beck and Haigh, 2014). We discuss here the intimate connection between these two areas of research and practice and use this connection as a basis to construct a novel model of personality.

Personality can be defined as the fairly stable psychological characteristics of an individual (McCrae and Costa, 2003; Corr and Matthews, 2009; Engler, 2013; Beck et al., 2015; Friedman and Schustack, 2015), which, according to a cognitive approach, are critically determined by the individual’s core knowledge – a set of core beliefs (Young, 2002; Dweck, 2008; Perdighe and Mancini, 2010; Beck et al., 2015; Osmo et al., 2018). This perspective, of course, raises two questions: (1) What are these core beliefs? (2) How are they acquired? With this respect, a particularly interesting aspect of attachment theory concerns the relationship between attachment and personality (Kobak, 1994; Carver, 1997; Hart, 2008; Lorenzini and Fonagy, 2013; Chiesa et al., 2017). As to question (2), the theory proposes that the core beliefs that make up one’s personality are formed through the relationship with one’s caregiver during childhood (Main et al., 1985; Platts et al., 2002; Sherman et al., 2015; Hesse, 2016; Marvin et al., 2016). Furthermore, according to the theory, this core knowledge involves three fundamental dimensions – disorganization, avoidance, and ambivalence (Bowlby, 1969/1982, 1973, 1980; Liotti, 2009; Paetzold et al., 2015; Feeney, 2016; Mikulincer and Shaver, 2016; Thompson, 2016). For example, the child who has a relationship with a cold caregiver develops the belief of being an unlovable person, while the child who has a relationship with a warm caregiver develops the belief of being a lovable person. These different beliefs are implicit, non-verbal, knowledge, which correspond to the different internal states the child can experience while relating to their caregiver in the avoidant dimension.

In this article, we argue that attachment-based approaches to personality are on the right track but need to be further expanded and elaborated upon. We propose four such amendments. First, we show that the nature of two of the three attachment dimensions described by attachment theory – avoidance and ambivalence – needs to be better specified. Second, we argue that four additional dimensions – phobicity, depressivity, somaticity, and obsessivity – are required to explain personality. Third, we propose a novel way to identify the caregiving features that induce the acquisition of each attachment dimension. Finally, we put forward an account of the specific mechanism through which attachment information is acquired: we argue that it is imprinting – a learning process evolutionarily preordained to be first performed in early sensitive periods – that gives attachment dimensions the quality of stable personality traits. In a nutshell, we propose a novel attachment-based model of personality, according to which the core knowledge underlying personality is made up by seven pieces of information imprinted through attachment interactions in response to certain caregiving features. We call this seven-dimensional Attachment Personality Model ‘APM.’

We will proceed as follows. In section one, we present the standard attachment view of personality. In section two, we illustrate how, despite its explanatory power, this view faces three major problems concerning personality, which involve: (1) stability, (2) intergenerational transmission, and (3) psychopathology. In section three, we argue that these three major problems faced by attachment theory can be solved if we supplement the theory by considering – overall – seven attachment dimensions that are induced by seven caregiving features and (unconsciously) acquired through imprinting. Importantly, we present five evidence-based arguments in favor of imprinting as the specific attachment acquisition mechanism. Finally, in section four, we discuss the implications and limitations of the proposed model.

Three caveats before we get started. Personality is a complex and controversial concept (Engler, 2013; Friedman and Schustack, 2015). We focus on the aspects of personality concerning acquired knowledge not because we think that this body of knowledge exhausts the nature of personality—we are well aware that genetical, biological, environmental, social, and cultural factors play a fundamental role in the constitution of personality—but simply because we think that core beliefs are a central *part* of personality and call for an explanation. In particular, by focusing on the *acquired information* underlying personality, our model can partially explain some of the differences in personality that do not arise from biological variables. We will therefore consider the influence of acquired information assuming a constant healthy biological substrate. Moreover, we do not suggest attachment knowledge to be the only acquired information that constitutes personality, but rather that it has a central role, given the early acquisition and crucial adaptation value.

Second, even though we situate our discussion at the interface of attachment theory and cognitive psychology, we consider our considerations completely compatible with psychoanalysis and most other clinical approaches. Finally, this article is mainly theoretical in scope: its aim is to develop a novel model which hasn’t be *directly* tested yet. Importantly, however, we intend to show that our proposal is *indirectly* supported by a large amount of evidence gathered from a variety of fields and is empirically *productive*, in that it generates a number of testable hypotheses.

## Attachment Theory

In this section, we outline the main features of attachment theory – the standard version of it, as it is currently broadly accepted – and its view of personality.

Bowlby (1969/1982, 1973, 1980), father of attachment theory, maintained that attachment is the fundamental evolutionary mechanism that every human being is provided with – *”from the cradle to the grave”* (Bowlby, 1969/1982, p. 208) – to obtain protection and care from a conspecific – a caregiver. For several years after birth, children need to be protected and cared for to survive and develop, and they are therefore inherently motivated to attach to a caregiver. Most often, the mother plays this fundamental role. However, despite the primacy of this early relationship, attachment remains prominent throughout life, and new attachment relationships with similar characteristics can be created at any age (Bowlby, 1969/1982; Allen, 2008; Kerns and Brumariu, 2016; Marvin et al., 2016; Mikulincer and Shaver, 2016; Fraley and Roisman, 2019). For example, when feeling vulnerable, an adult will look for protection and care from their partner as a child does from their caregiver, although usually in a different form. While the child cries and clings, the adult might call their loved one and relate a difficult day at work looking for sympathy.

According to attachment theorists, a fundamental aspect related to the formation of attachment relationships is the knowledge acquired through such relationships. This acquired knowledge—attachment theory also says—is a fundamental part of personality. Let us consider these two points in turn.

### Attachment-Knowledge and Internal Working Models

Attachment knowledge has been described through the concept of Internal Working Models (IWMs), in which specific representations of the caregiver and the self—and hence of the attachment-caregiving relationship—are generated and stored (Bowlby, 1973; Bretherton and Munholland, 2008; Sherman et al., 2015; Marvin et al., 2016). We consider here these models as a whole and refer to them as the IWM. Attachment representations are taken to consists of three dimensions: disorganization, avoidance, and ambivalence (Bartholomew and Horowitz, 1991; Brennan et al., 1998; Fraley and Spieker, 2003; Liotti and Farina, 2011; Fraley et al., 2015; Paetzold et al., 2015; Mikulincer and Shaver, 2016). We call them ‘α-dimensions.’ All dimensions are acquired by each individual in different degrees. They are generally considered reciprocally (statistically) independent, although some correlation between avoidance and ambivalence has been found measuring adult attachment (Cameron et al., 2012; Fraley et al., 2015). An individual who is neither avoidant nor ambivalent is referred to as being secure. Otherwise stated, who is either avoidant or ambivalent is considered to be insecurely attached.

Attachment information is primarily represented at an implicit, non-verbal, level (Bowlby, 1969/1982; Schore, 2000), and can therefore be assessed only indirectly. Classical ways to do that are the Strange Situation Procedure (SSP) (Ainsworth et al., 1978; Main and Solomon, 1990) – a lab procedure to measure attachment behaviors in infants – and the Adult attachment interview (AAI) (George et al., 1985; Main and Hesse, 1990; Hesse, 2016) – a structured interview to evaluate the state of mind with regards to attachment in adults. Through these instruments, four different attachment styles – disorganized, secure, avoidant, and ambivalent – can be measured. We want to point out that we will use here the term ‘style’ in a proprietary way, focusing on attachment representations and dimensions. Attachment theory is complex, and researchers with different orientations tend to use different terms, which imply their focus on different aspects of attachment. In particular, attachment ‘pattern’ and ‘state of mind’ traditionally belong to the developmental area and ‘style’ to the social one. It is also worth noting that instruments developed in different areas, such as the AAI and adult attachment questionnaires, have shown minimal correlation (Crowell et al., 2016). These instruments differ in many respects, but the AAI – originally developed as a categorical tool – has then been found to be underpinned by dimensions (Roisman et al., 2007), like the questionnaires, thereby further supporting the dimensional perspective endorsed by this work.

Given the implicit nature of the fundamental attachment knowledge, its identification can be performed only indirectly and can be synthetized as follows. Disorganization is a representation of the caregiver’s frightfulness. In this case, the IWM represents the caregiver as being both a source of protection and a threat for the child, thereby leading to disorganized behaviors (Main and Solomon, 1990; Liotti, 2004, 2011; Lyons-Ruth and Jacobvitz, 2016). Avoidance is a representation of the caregiver’s loving attitude toward the child and their sensitivity to the child’s emotional life, while ambivalence represents the caregiver’s availability and reliability (Ainsworth et al., 1978; George et al., 1985; Hesse, 2008; Mikulincer and Shaver, 2016). As a result, an avoidant child expects their caregiver not to be interested in their inner life, and an ambivalent child expects their caregiver not to be there for them in case of need. Notably, each attachment style is primarily characterized by (implicit) information, which manifests itself in both behaviors and internal states. Given that an individual can appear to be more or less disorganized, avoidant, or ambivalent, the stored information has a clear continuous nature.

Attachment researchers agree that the represented α-dimensions crucially depend on the relation between the child and the caregiver. In particular, an insecure child’s representation of their caregiver is taken to be strongly related to the caregiver’s sensitivity – defined as how adequately and promptly the caregiver detects and satisfies the child’s needs (Ainsworth et al., 1978; De Wolff and van Ijzendoorn, 1997). Given our purposes, a crucial aspect for the information stored in the IWM is that it appears to be present across the lifespan and to manifest itself in one’s adult relations (Ainsworth et al., 1978; Parkes et al., 1993; Green et al., 2000; Fraley and Shaver, 2008; Mikulincer and Shaver, 2016). In other words, this information is the latent foundational part of the *attachment styles* that manifest themselves at any age (Ammaniti et al., 2000; Target et al., 2003; Stievenart et al., 2012). An avoidant adult tends to be dismissing and detached in their romantic relationships as much as an avoidant child tends to be over-autonomous and unemotional with their caregiver.

We will argue that attachment knowledge is not only present during the entire course of life but also stable, by virtue of its specific acquisition mechanism – imprinting. Bowlby (1969/1982) first indicated that the attachment of a child to their caregiver is realized through imprinting in an early sensitive period of life – that usually ends within the sixth month. After attaching to a specific caregiver, the child needs to acquire the information to build their IWM and dimensions. Such information – that allows them to best adapt to their caregiver – needs to be acquired within the first 24 months, otherwise they will suffer from terrible psychological dysfunctions (Marvin et al., 2016; Troller-Renfree and Fox, 2017). The period between 6 and 24 months can therefore be considered as sensitive for the acquisition of the α-dimensions – disorganization, avoidance, and ambivalence.

The fact that attachment knowledge persists across one’s life brings us to the second issue: the relation between attachment and personality.

### Attachment-Knowledge and Personality

*“From the viewpoint of the position adopted, adult personality is seen as a product of an individual’s interactions with key figures during all his years of immaturity, especially of his interactions with attachment figures.”* (Bowlby, 1973, p. 208)

Although usually attachment theorists do not define personality explicitly, they recognize the strict relationship between attachment and personality, which have appeared evident since the foundation of the theory and have been confirmed by numerous studies (Bowlby, 1969/1982, 1973, 1980; Guidano, 1987, 1991; Noftle and Shaver, 2006; Guidano, 2007; Chopik et al., 2013; Levy et al., 2015; Mikulincer and Shaver, 2016; Karterud and Kongerslev, 2019; Rosa-Mendes et al., 2019; Young et al., 2019). Such relationship is so tight that attachment styles are often implicitly taken as being themselves personality characteristics (Bowlby, 1973; Mikulincer and Shaver, 2012).

At least four areas of attachment research touch on this subject. (1) The quality of early attachment has been found to have profound developmental consequences, for example, in terms of emotional regulation, self-esteem, resilience, social attitude, and competence (Sroufe, 2005; Thompson, 2016). In particular, emotional regulation appears to be directly related to attachment styles (Viddal et al., 2017; Mikulincer and Shaver, 2019). (2) In their romantic relationships, secure, avoidant, and ambivalent adults adopt specific strategies, being the secure considerably more adaptive compared to insecure ones – in terms of both personal satisfaction and relationship functioning (Fraley et al., 2011; Mikulincer and Shaver, 2016). (3) A great number of studies demonstrates the link between disorganized/insecure attachment and psychopathology, in particular personality disorders (Lorenzini and Fonagy, 2013; Stovall-McClough and Dozier, 2016; Chiesa et al., 2017). For example, convincing arguments and evidence show the causal role of early disorganization of attachment in the development of a borderline personality disorder (Liotti and Farina, 2011). (4) Finally, attachment styles are connected to mating strategies, which strongly characterize adult behavior (Chisholm, 1996; Simpson and Belsky, 2016).

The connection between attachment and personality emerges with particular clarity from the TAM (Temperament-Attachment-Mentalizing) model of personality elaborated by Karterud and Kongerslev (2019). These authors effectively synthesize the complexity of personality by identifying in T, A, and M the three major components of personality. Temperament is the innate biological basis of personality, attachment is what is learned early in life from the caregiver, and mentalizing is a higher-level cognitive ability grounded in attachment and developed throughout life. Therefore, according to the TAM model, attachment can be considered the fundamental acquired knowledge that constitutes personality.

## Attachment Theory: Three Major Problems Concerning Personality

Notwithstanding its great achievements, attachment theory is widely recognized to be faced with three major general problems. In this section, we show how these three issues represent a problem also when trying to account for the relationship between attachment and personality – despite the clear link discussed above.

### Problem P1: Intergenerational Transmission

A central issue in attachment theory is the intergenerational transmission of attachment styles. Both attachment and clinical sources support the style transmission from a generation to the next (Bowlby, 1969/1982, 1973, 1980; Guidano and Liotti, 1983; Guidano, 1987, 1991; Bretherton, 1993; Van Ijzendoorn, 1995; Bernier et al., 2014; Sette et al., 2015; Verhage et al., 2016; Van Ijzendoorn and Bakermans-Kranenburg, 2019). According to this phenomenon, a child will most likely acquire the style of their main caregiver. By measuring the child’s style through the SSP and that of their caregiver through the AAI, most likely, we will have: (A) An avoidant child from an avoidant caregiver; (B) A secure child from a secure caregiver; and (C) An ambivalent child from an ambivalent caregiver. However, the identification of the caregiving features responsible for bringing about each attachment dimension remains an open problem. An extensive statistical analysis indicates caregiver’s sensitivity as not being the only cause of avoidance and ambivalence (Van Ijzendoorn, 1995; Verhage et al., 2016; Van Ijzendoorn and Bakermans-Kranenburg, 2019), and other caregiving features have been proposed to fill this so-called transmission gap (Whipple et al., 2011; Bernier et al., 2014; Van Ijzendoorn and Bakermans-Kranenburg, 2019). In other words, although sensitivity is the feature that is supposed to be involved in fostering attachment security, being sensitive seems not to be enough to raise a secure child. The standard theory has proven unable to fill this gap with its own means. As a result, the origin of attachment-personality remains uncertain.

### Problem P2: Stability

Personality is (relatively) stable by definition. Therefore, if attachment is – as generally accepted – a fundamental part of personality, then attachment should also be (relatively) stable. This means that an individual’s attachment style over time should – in general – stay the same. According to this view, an avoidant child, for example, is expected to become an avoidant adult. However, this crucial aspect of attachment remains controversial: attachment studies that investigated its consistency across the lifespan found different results and, often, only modest stability (Waters et al., 2000; Fraley, 2002; McConnell and Moss, 2011; Pinquart et al., 2013; Kobak et al., 2016). In other words, according to the standard view, we face a contradiction: attachment is supposed to be, at the same time, a central part of personality and not really stable – while personality is. The standard theory seems to be caught in an unsolvable dilemma.

### Problem P3: Psychopathology

Finally, the link between attachment and psychopathology is unanimously recognized. In particular, disorganization has been identified as a cause of dissociative pathologies (Liotti, 1992, 2004; Liotti and Farina, 2011; DeKlyen and Greenberg, 2016; Lyons-Ruth and Jacobvitz, 2016; Mikulincer and Shaver, 2016; Stovall-McClough and Dozier, 2016), and avoidance and ambivalence as generally correlated to most mental disorders (DeKlyen and Greenberg, 2016; Stovall-McClough and Dozier, 2016). A frightening caregiver favors the development of dissociative symptoms – such as depersonalization and derealization (i.e., an alteration of the individual’s perception of themselves or the world around respectively) – while an insensitive caregiver characterizes the childhood of most people who suffer, as an adult, from a common mental disorder – such as mood, anxiety, eating, obsessive disorders – suggesting a profound and durable effect of early attachment on the individual. Given its implications on a personal and social level, this problem is the most critical one. In fact, it has been accurately taken into account, and many clinicians believe that attachment should play a primary role in the therapeutic process (Obegi and Berant, 2010; Berry and Danquah, 2016). However, the underlying factors that connect insecure attachment to psychopathology remain unidentified. Again, the standard theory is stuck. It could only prove the implication of attachment in psychopathology with no further specification.

### Attachment Theory Is in Trouble: What Should We Do?

Standard attachment theory identifies attachment as a fundamental part of personality. However, the theory confronts three critical problems that challenge the validity of this claim. (1) Sensitivity – the caregiving feature indicated as involved in the early attachment relationship – is not sufficient to explain the acquisition of the attachment knowledge by the child. (2) Attachment cannot be simultaneously a central part of personality and relatively unstable over life because personality is stable. (3) Psychopathology is deeply affected by attachment, but the theory is unable to specify how exactly.

These problems suggest two possibilities: (1) Attachment is not really a central part of personality; or (2) Attachment is a central part of personality, but we need to enhance the theory in order to fully account for this centrality. In the following sections, we show how the second option might well be the valid one and its remarkable implications.

## Attachment Dimensions and the Imprinted Personality

This section is dedicated to outlining the APM enhancement of standard attachment theory to account for the relationship between attachment and personality. We start our argumentation assuming (in sections “α-Dimensions: The Three Basic Attachment Dimensions” and “β-Dimensions: The Four Additional Attachment Dimensions”) imprinting as the specific mechanism of attachment knowledge acquisition. Although the standard theory implicitly accepts imprinting as such a mechanism, it has never elaborated on the concept, and imprinting and its implications have never been adequately considered. Therefore, we then provide (in section “Imprinting and Sensitive Periods”) detailed arguments that support imprinting. Overall, we suggest that seven dimensions are first imprinted over two consecutive early sensitive periods:

1. Three α-dimensions – disorganization, avoidance, and ambivalence – over an α-period (between 6 and 24 months). As mentioned above, this has already been found by the standard theory.
2. Four β-dimensions – phobicity, depressivity, somaticity, and obsessivity – over a β-period (between 2 and 6 years). This is an APM proposal.

Although we focus here on the dimensions, the attachment to a specific caregiver is also realized through imprinting and is usually already accomplished within the first 6 months of life (Bowlby, 1969/1982; Marvin et al., 2016; Table 1).

**TABLE 1:** Sensitive periods and imprinting.

	**Acquisition process**	**Period**	**Object**
0	Imprinting	2–6 months	Caregiver’s Identity
1	Imprinting	α-Period: 6–24 months	α-Dimensions
2	Imprinting	β-Period: 2-6 years	β-Dimensions

*Three consecutive sensitive periods for attachment are indicated. (1) Between 2 and 6 months, the identity of the caregiver is usually already acquired, but the sensitive period extends to 24 months. (2) The α-Period (6–24 months) is the sensitive period for the α-dimensions – disorganization, avoidance, ambivalence. Currently, 24 months is considered to be the maximum extensions of the attachment sensitive period. (3) The β-Period (2–6 years) is the sensitive period for the β-dimensions – phobicity, depressivity, somaticity, obsessivity. This additional period is proposed by the APM. Beginning and end of each period are approximate, and clear-cut demarcations are indicated for simplicity.*

According to our proposal, seven imprinted dimensions fully account for the centrality of attachment in personality. In particular, we argue that: (1) A redefinition of avoidance and ambivalence allows the APM to solve the intergenerational transmission gap (solution to problem P1, section “α-Dimensions: The Three Basic Attachment Dimensions”); (2) The introduction of four additional dimensions allows the APM to fully explain the causal relationship between attachment and psychopathology (solution to problem P3, section “β-Dimensions: The Four Additional Attachment Dimensions”); (3) Imprinting accounts for the stability of attachment and, therefore, personality (solution to problem P2, section “Imprinting and Sensitive Periods”). Importantly, this mechanism, although ensuring general stability, also allows for change at any age.

### α-Dimensions: The Three Basic Attachment Dimensions

According to the standard theory, the three α-dimensions – disorganization, avoidance, and ambivalence – are first acquired during the α-period, which corresponds to most infancy (6-24 months).

Disorganization corresponds to the collapse of any organized strategy under enough stress and has been clearly linked to the experience of a frightening caregiver, which generates a ‘fear without solution’ (Main and Hesse, 1990; Main and Solomon, 1990). Such a caregiver can be perceived as directly or indirectly threatening. An important case of indirect threat for a child is a caregiver who is suffering from an ‘unresolved state of mind with respect to loss or trauma’ – a condition that is also more easily found in adults who were disorganized children (Lyons-Ruth and Jacobvitz, 2008; Liotti and Farina, 2011). Disorganized experiences have a profound impact on personality, as suggested by their connection with dissociative symptoms and psychopathology. Disorganization typically leads to strategies that aim to reach some form of organization. Such strategies can hide disorganization but often correspond to dysfunctional conditions, as further discussed in section “Implications for Psychopathology.” On the other hand, the connection of avoidance and ambivalence to caregiving features is still controversial with a transmission gap that remains to be filled.

#### A New Definition of Avoidance and Ambivalence

The APM proposes that the α-dimensions derive each from one corresponding caregiving feature (Table 2), which we term α-features. To account for such a causal link, we suggest the following – more specific – definition of avoidance and ambivalence starting from a precise definition of the corresponding α-features – that we refer to as sensitivity and responsiveness respectively:

**TABLE 2:** Caregiving features and corresponding attachment dimensions.

	**α-Feature**		**α-Dimension**
1	Frightening caregiver	⟹	Disorganized child
2	Insensitive caregiver	⟹	Avoidant child
3	Unresponsive caregiver	⟹	Ambivalent child

	**β-feature**		**β-dimension**

4	Limiting caregiver	⟹	Phobic child
5	Unreachable caregiver	⟹	Depressive child
6	Defining caregiver	⟹	Somatic child
7	Judgmental caregiver	⟹	Obsessive child

*The APM proposes seven attachment dimensions that derive each from a specific caregiving feature. Three α-features induce the three α-dimensions, and four β-features induce the four β-dimensions. For example, an insensitive caregiver induces avoidance in their child, a defining caregiver induces somaticity in their child. We show how, primarily, each β-dimension provides adaptation to a specific β-feature, and the four fundamental adaptation problems they address are particularly salient in the β-period.*

• **Sensitivity:** the emotional connection offered by the caregiver. Sensitivity is the feature of love, the ‘emotional warmth,’ which is communicated by the caregiver to the child.

**Responsiveness:** the physical availability offered by the caregiver. Responsiveness is the feature of ‘being physically there when needed.’

• **Avoidance:** the subjective measure of the caregiver’s insensitivity. The more the caregiver is unloving, the higher is avoidance.

**Ambivalence:** the subjective measure of the caregiver’s responsiveness. The more the caregiver is physically unavailable when needed, the higher is ambivalence (for the child, the caregiver should and could be there but is not).

These definitions entail a two-channel-hypothesis – i.e., that two main (relatively) independent communication channels, one emotional and the other physical, are first relevant in the attachment relationship – with a one-to-one causal link between caregiving features and attachment dimensions:

1. Emotional-Channel. Sensitivity affects avoidance, and only avoidance: sensitivity is the emotional α-feature and avoidance the emotional α-dimension.
2. Physical-Channel. Responsiveness affects ambivalence, and only ambivalence: responsiveness is the physical α-feature and ambivalence the physical α-dimension.

The insensitive caregiver does not engage emotionally and encourages the child to deactivate attachment. As a result, the child tends to avoid interactions based on attachment. The unresponsive caregiver does not engage physically and encourages the child to hyper-activate attachment. As a result, the child tends to be worried about the caregiver’s availability (with evident emotional display).

Importantly, these definitions provide a clear correspondence between caregiving features and attachment dimensions. Insensitivity and avoidance match with each other by virtue of their emotional nature. The avoidant avoids the intolerable experience of facing a cold caregiver by deactivating attachment and focusing on other activities (Ainsworth et al., 1978; George et al., 1985; Hesse, 2008; Mikulincer and Shaver, 2016). A child will typically explore or play. When the insensitive caregiver is present, they do not connect emotionally and, as a result, asking comfort from them makes no sense. Unresponsiveness and ambivalence match with each other by virtue of their physical nature. The ambivalent hyper-activates attachment and worries whether their caregiver will be physically there for them (Ainsworth et al., 1978; George et al., 1985; Hesse, 2008; Mikulincer and Shaver, 2016). The unresponsive caregiver is unpredictably available but connects emotionally when present and, as a result, an emotional signal demanding their presence makes perfect sense. Interestingly, an insensitive-unresponsive caregiver will correspond to an avoidant-ambivalent child, but the two dimensions cannot be expressed simultaneously (since deactivation and hyper-activation are incompatible).

Hence, the provided definitions perfectly illustrate the basic characteristics of the attachment relationship. Their adequacy is further supported by the following four arguments.

(1) **Statistical-independence argument.** As discussed above, avoidance and ambivalence as defined in the literature are generally considered mutually (relatively) independent. We define avoidance and ambivalence more specifically – referring to the caregiver’s sensitivity and responsiveness – but still maintain that they are (relatively) independent. In fact, if they are, there must be two mutually (relatively) independent caregiving features, each of which induces one of them, and they also need to be identified.

The definitions given above support independence both between insensitivity and unresponsiveness and between avoidance and ambivalence. Indeed:

1. One is an emotional variable (insensitivity/avoidance) and the other a physical one (unresponsiveness/ambivalence).
2. Any combination of the emotional and physical variables is possible. In particular, a caregiver can independently provide any degree of emotional and physical care. For example, as emerges from a broad range of research (Bowlby, 1969/1982, 1973, 1980; Ainsworth et al., 1978; Guidano and Liotti, 1983; Guidano, 1987, 1991; Schore, 1994; Sroufe, 1995; Guidano, 2007; Hesse, 2008; Mikulincer and Shaver, 2016), a caregiver can be physically there but emotionally disconnected and, conversely, they can usually be not physically there but emotionally connected when present.

The given definitions meet the requirement of reciprocal (relative) independence. By contrast, the literature has not focused on the mutual independence of caregiving features. Distinguishing between a purely emotional channel and a purely physical one allows us to ensure such independence.

(2) **Developmental argument.** We consider two points. (A) In order to develop adequately, for the child, both emotional connection and physical care are essential (Bowlby, 1969/1982, 1973, 1980; Stern, 1985; Schore, 1994; Sroufe, 1995; Leerkes and Wong, 2012; Marvin et al., 2016; Feldman, 2017). (B) As mentioned above, some caregiving features have been suggested as inducing avoidance and ambivalence. In particular, Bernier et al. (2014) found that sensitivity and autonomy support as defined by Whipple et al. (2011, p. 397), although not reciprocally independent, fully explain the generation of avoidance and ambivalence. This entails that it is possible to find two caregiving features on which avoidance and ambivalence entirely depend.

These data suggest that we should seek the cause of avoidance and ambivalence considering (A) the emotional and physical aspects of development and (B) two caregiving features. This is, indeed, the content of our proposal.

(3) **Evolutionary argument.** We have reason to believe that in the Environment of Evolutionary Adaptedness (EEA), the context of human evolution (Bowlby, 1969/1982), avoidance and ambivalence have been transmitted through two mutually independent channels – one emotional and the other physical. More precisely, according to an evolutionary argument, avoidance derives from the unwillingness to invest and ambivalence from the inability to invest in the offspring (Chisholm, 1996; Chisholm and Sieff, 2014).

On the one hand, unwillingness is an emotional feature that expresses intentional rejection. Evolutionarily, this is due to a harsh environment that makes the caregiver opt for investing in mating as opposed to parenting in order to maximize their reproductive success. In this condition, the child finds the best fit ignoring their caregiver and boosting their autonomy (avoidance).

On the other, inability is a physical feature that expresses the impossibility to be physically there. Evolutionarily, this is due to an unpredictable environment that occasionally forces the caregiver to attend to essential survival activities, thereby abdicating their role. In this condition, the child finds the best fit amplifying their need signals (ambivalence).

The evolutionary argument is supported by developmental evidence. Indeed, caregiver willingness and ability seem to be crucial information for young children as proved by the ability of 9-month-old infants to distinguish between the unwillingness and inability of an adult to hand them a toy (Behne et al., 2005).

(4) **Neuroscientific argument.** We have defined: (A) Insensitivity and avoidance as socio-emotional properties. As such, they primarily rely on gaze direction and facial expressions (relevant to emotional connection). (B) Unresponsiveness and ambivalence as socio-physical properties. As such, they primarily rely on reciprocal position (relevant to physical protection and care).

Two quite independent brain networks can be identified as underpinning these emotional and physical aspects. (A) On the one hand, the superior temporal sulcus, which is essential to detect gaze direction (Pelphrey et al., 2005; Hoehl et al., 2009; Itier and Batty, 2009; Carlin and Calder, 2013), is connected to the amygdala, which is key to reading facial emotions (Loughead et al., 2008; Whalen et al., 2013; Gothard, 2014; Wang et al., 2017). (B) On the other, the precuneus region is essential to deem the reciprocal position (Peer et al., 2015).

These neuroscientific data are consistent with two (relatively) independent channels, one emotional and the other physical, that underpin insensitivity/avoidance and unresponsiveness/ambivalence respectively.

Concluding this section, we want to stress that the focus on these two basic channels is proposed to help further understand and differentiate the complex nature of the two fundamental dimensions of avoidance and ambivalence and not to reduce it to such channels. In other words, the two channels are suggested to catch a fundamental distinction between the two dimensions and not to describe them exhaustively.

#### Solution to the ‘Intergenerational Transmission Problem’ (P1)

Overall, the above arguments converge to strongly support the two-channel-hypothesis. With these definitions, three attachment dimensions and three corresponding caregiving features are revealed to be clearly causally related: (1) a frightening caregiver induces disorganization, (2) an insensitive caregiver induces avoidance, and (3) an unresponsive caregiver induces ambivalence. Accordingly, each α-dimension can be thought of as corresponding to a core belief: “My caregiver is frightening,” “My caregiver is insensitive,” and “My caregiver is unresponsive” respectively (Table 3). Therefore, the APM indicates how attachment – and the related aspects of personality – are transmitted from a generation to the next, thereby solving the intergenerational transmission problem (P1). However, three dimensions are still insufficient to account for the relationship between attachment and psychopathology – we suggest these dimensions to be seven.

**TABLE 3:** Attachment dimensions as core beliefs.

	**Dimension**	**Core belief**	**Definition: Subjective measure of how much the caregiver is**
α-Dimensions	1 Disorganization	“My caregiver is frightening”	Frightening
	2 Avoidance	“My caregiver is not going to love me”	Insensitive
	3 Ambivalence	“My caregiver is usually not available”	Unresponsive
β-Dimensions	4 Phobicity	“I am in danger if my caregiver is not with me”/“My caregiver won’t let me go”	Limiting
	5 Depressivity	“I won’t be able to reach my caregiver emotionally”	Unreachable
	6 Somaticity	“I need my caregiver to tell me about myself”/“My caregiver will intrude on me”	Defining
	7 Obsessivity	“I am wicked”	Judgmental

*According to the APM, the IWM of attachment consists of seven dimensions that are first imprinted in sensitive periods early in life. Each dimension (1) corresponds to a core belief – a very simple but evolutionarily valuable piece of information – and (2) is defined as the subjective measure of the caregiving feature by which it is induced.*

### β-Dimensions: The Four Additional Attachment Dimensions

Analogously to the α-case, we propose a β-period corresponding to the preschool years (2–6 years) as sensitive for the imprinting of four β-dimensions – phobicity, depressivity, somaticity, and obsessivity (Table 1) – and that these dimensions also derive each from one corresponding caregiving feature, which we term β-features (Table 2). Indeed, as we discuss below, the caregiver can be:

1 **Limiting:** The caregiver regulates the child’s balance between attachment and exploration. When the caregiver is exploration-limiting, they induce a sense of vulnerability in the child. The attachment consequence is **phobicity**.
2 **Unreachable:** The caregiver should be emotionally reachable for the child. When the caregiver is emotionally unreachable, they induce a sense of defeat in the child. The attachment consequence is **depressivity**.
3 **Defining:** The caregiver regulates the child’s internal states, in particular sensations and emotions, and supports the child’s own definition of them. When the caregiver imposes their own definitions to the child, they induce a sense of somatic uncertainty in the child. The attachment consequence is **somaticity**.
4 **Judgmental:** The caregiver provides an ethical reference to the child. When the caregiver is judgmental to the child, they induce a sense of being wicked in the child. The attachment consequence is **obsessivity**.

Importantly, these features are indicated as the principal causes of the corresponding dimensions without excluding other possible influences on them. Moreover, we suggest that the β-dimensions are the typical cause of specific mental disorders. Indeed, analyzing vast clinical samples of patients suffering from the most common mental disorders – such as anxiety, mood, eating, and obsessive disorders – Guidano and Liotti found that these patients organized their pathological knowledge following four patterns – or ‘cognitive organizations’ (CO) (also called ‘personal meaning organizations’) (Guidano and Liotti, 1983; Guidano, 1987, 1991, 2007; Table 4).

**TABLE 4:** β-Dimensions and corresponding cognitive organizations.

	**β-Dimension**	**Corresponding cognitive organization (CO)**	**References**
1	Phobicity	Phobic CO	Guidano and Liotti, 1983, pp. 221–227; Guidano, 1987, pp. 139–154; Guidano, 1991, pp. 41–45; Guidano, 2007, pp. 79–88
2	Depressivity	Depressive CO	Guidano and Liotti, 1983, pp. 190-193; Guidano, 1987, pp. 124—38; Guidano, 1991, pp. 35–40; Guidano, 2007, pp. 62–78
3	Somaticity	Eating disorder CO	Guidano and Liotti, 1983, pp.291–294; Guidano, 1987, pp. 155–171; Guidano, 1991, pp. 45–50; Guidano, 2007, pp. 88–103
4	Obsessivity	Obsessive–compulsive CO	Guidano and Liotti, 1983, pp. 261–266; Guidano, 1987, pp. 172–187; Guidano, 1991, pp. 50–56; Guidano, 2007, pp. 103–114

*The β-dimensions correspond each to a ‘cognitive organization’ (also referred to as ‘personal meaning organization’). The references indicate where a detailed description of such organizations can be found.*

According to their studies, such COs favor the onset and maintenance of specific disorders – and, therefore, can be considered their cause. This research has been then confirmed by Guidano’s followers and extensively tested in clinical practice (Nardi and Bellantuono, 2008; Arciero and Bondolfi, 2009; Nardi et al., 2010). Furthermore, tools have been conceived to assess the four COs both in healthy and pathological conditions (Picardi et al., 2003; Nardi et al., 2012). Essentially, the organizations of knowledge identified by Guidano and Liotti can be considered higher-level descriptions of personality traits, and their careful analysis allows for the extraction of characterizing core beliefs that evidently correspond to those implied by the β-dimensions (Table 3). In other words, the β-dimensions can be considered the foundation of the COs (Guidano and Liotti, 1983; Guidano, 1987, 1991, 2007; Nardi et al., 2010), and, therefore, such dimensions result to be causally related to psychopathology (Table 5).

**TABLE 5:** Attachment dimensions that can be a cause of a mental disorder.

	**Dimension**	**Main causally related mental disorders**
1	Disorganization	Dissociative disorders
2	Phobicity	Separation anxiety, agoraphobia, and panic disorder
3	Depressivity	Depression
4	Somaticity	Eating disorders
5	Obsessivity	Obsessive–compulsive disorder

*Given their characteristics, five of the seven attachment dimensions – disorganization, phobicity, depressivity, somaticity, and obsessivity – are causally related to psychopathology. Standard attachment theory recognizes the role of disorganization, while the studies of Bowlby, Liotti, and Guidano and his followers prove the role of phobicity, depressivity, somaticity, and obsessivity. These dimensions can cause the onset and maintenance of a specific mental disorder by making the subject more sensitive to conditions that favor it.*

Finally, we want to stress that the β-dimensions belong to every personality and, although they are connected to mental disorders, a high level of such dimensions does not entail psychopathology. They can be considered personality traits, whose excessive presence can facilitate a related disorder. Therefore, being phobic, depressive, somatic, or obsessive does not imply any pathology (and can actually favor adaptation). Similarly, a mental disorder typically related to these dimensions can have an etiology that is not related to them.

We outline each β-dimension in this section and provide evidence of a sensitive β-period in the next.

#### Phobicity and the Limiting Caregiver

We define phobicity as the subjective measure of how much the caregiver is limiting (in terms of the child’s exploration). The child needs to get their tendencies to attach and explore regulated by the caregiver, and the phobic child experiences a limitation of their exploration. In other words, the caregiver somehow forces the child to be closer to them than the child feels necessary. For example, the caregiver may be hyper-protective and tend to keep the child under their strict control. In this case, the child will tend to feel restricted and, when confronted with the task of autonomously exploring the environment, particularly in danger.

As a result, we propose, the preschool child gets imprinted an implicit knowledge that can be expressed by the core belief “I am in danger if my caregiver is not with me” and “My caregiver won’t let me go” (Table 3). This knowledge characterizes phobicity making the phobic particularly sensitive to the balance attachment-exploration and inclined to suffer from (1) separation anxiety when too far from the caregiver and (2) a sense of constriction when too close. In practice, phobicity is revealed by such sensitivity.

Our definition and proposal are supported by the following evidence.

##### Caregiving-attachment

The balance between protection and autonomy support is a fundamental caregiving task (Bowlby, 1973; Skinner et al., 2005; Bernier et al., 2014), and autonomy support has been found to be especially important in the preschool years for the following socio-emotional development (Matte-Gagné et al., 2015). The parental styles that induce phobicity have been found to be of two kinds, both of which result in limiting child exploration (Bowlby, 1973; Guidano and Liotti, 1983; Guidano, 1987, 1991, 2007): (A) Direct: the caregiver is over-protective; (B) Indirect: the caregiver makes the child fear to lose them if they do not stay close – for example, by complaining about a serious illness.

##### Clinical

The caregiver limitation of exploration, in any of its forms, is causally related to the main clinical manifestations of phobicity – separation anxiety, agoraphobia, and panic disorder (Bowlby, 1973; Guidano and Liotti, 1983; Guidano, 1987, 1991, 2007; Nardi et al., 2010). Indeed, despite the many intervening variables, the limitation of exploration suffered in childhood has been found correlated to adult panic disorder (Faravelli et al., 1991, 2010), and separation anxiety as a child to agoraphobia and panic attacks as an adult (Ayuso et al., 1989; Silove et al., 1995; Kossowsky et al., 2013).

##### Evolutionary

In a difficult EEA, the child that gets imprinted to stay close to the caregiver enhances their chances to survive, especially when they develop the ability of autonomous exploration (β-period, 2–6 years). Later, the phobic adult will tend to ensure they have their caregivers at hand, thereby improving their survival and reproductive chances in a harsh environment.

#### Depressivity and the Unreachable Caregiver

We define depressivity as the subjective measure of how much the caregiver is emotionally unreachable. The child needs to be able to reach the caregiver for emotional care, and the depressive child experiences a failure of their attempts to do so. In other words, the caregiver is, for some reason, emotionally unavailable to the child when the child tries to reach them. For example, the caregiver may be usually away from home. In this case, the child will tend to see their desire for emotional care frustrated and, therefore, to feel hopeless, defeated (Seligman, 1975; Guidano and Liotti, 1983; Guidano, 1987, 1991, 2007).

As a result, we propose, the preschool child gets imprinted an implicit knowledge that can be expressed by the core belief “I won’t be able to reach my caregiver” (Table 3). This knowledge characterizes depressivity making the depressive particularly sensitive to the loss of the caregiver and inclined to suffer from (1) a sense of defeat and (2) depression when a loss is perceived. In practice, depressivity is revealed by such sensitivity.

Our definition and proposal are supported by the following evidence.

##### Caregiving-attachment

Ensuring emotional availability to the child is a fundamental caregiving task (Bowlby, 1980; Skinner et al., 2005). The emotional reachability of the caregiver has been found to be especially important in childhood (Kendler et al., 2000; Otowa et al., 2013), and particularly in the preschool years (Belden et al., 2007), for the following socio-emotional development. A cold and demanding parental style (affectionless control) has been identified as connected to the development of future depression (Guidano and Liotti, 1983; Parker, 1983; Guidano, 1987, 1991, 2007). Moreover, the most evident depressive parental characteristic is the long physical absence, especially their loss, which clearly entails emotional unreachability (Beck, 1967; Brown and Harris, 1978; Bowlby, 1980; Guidano and Liotti, 1983; Slavich et al., 2011; Otowa et al., 2014). Neuroscientific research confirms the connection between early loss and future depression (Panksepp, 1998) and the preschool years as a sensitive period (Panksepp and Biven, 2012).

##### Clinical

The caregiver emotional unreachability is causally related to the main clinical manifestation of depressivity – depression (Bowlby, 1980; Guidano and Liotti, 1983; Guidano, 1987, 1991, 2007; Nardi et al., 2010). Accordingly, the lack of emotional reciprocity is considered to be a primary cause of the onset of depression in preschool children (Belden et al., 2007), and suffering long early separation or loss significantly increases the sensitivity to future loss as an antecedent to depression (Slavich et al., 2011).

#### Evolutionary

In a difficult EEA, the child that gets imprinted to give up attempting to reach an emotionally unavailable caregiver enhances their chances to survive by becoming self-reliant (Bowlby, 1980). A tendency to self-reliance starts to be feasible when the child begins to develop some independence (β-period, 2–6 years). Later, the depressive adult will have the advantage to be used to relying on themselves, thereby improving their survival and reproductive chances in a harsh competitive environment.

#### Somaticity and the Defining Caregiver

We define somaticity as the subjective measure of how much the caregiver is defining (in terms of the child’s internal states). The child needs to learn to recognize their internal states – primarily, the most somatic ones: sensations and emotions – from the caregiver, and the somatic child experiences, instead, an external definition. In other words, the caregiver somehow forces the child to adopt an internal state that does not match the child’s actual one. For example, the child might be suggested they feel pain when they do not (like in the case of Brenda, Guidano, 1987). In this case, the child will tend to feel confused about their own state and intruded upon by the caregiver. Somaticity is, therefore, an anomaly of the fundamental caregiving regulation of the child’s internal states, where the caregiver’s state does not correspond to the child’s state. This way, the child does not learn how to recognize their own internal states and becomes uncertain of them.

As a result, we propose, the preschool child gets imprinted an implicit knowledge that can be expressed by the core belief “I need my caregiver to tell me about myself” and “My caregiver will intrude on me” (Table 3). This knowledge characterizes somaticity making the somatic particularly sensitive to the definition of their own internal states and inclined to suffer from (1) uncertainty when not sufficiently defined and (2) a sense of intrusion when being excessively defined by the caregiver. In practice, somaticity is revealed by such sensitivity.

Our definition and proposal are supported by the following evidence.

##### Caregiving-attachment

The regulation of the child’s internal states is a fundamental caregiving task performed through behavioral and biological synchronicity between caregiver and child (Stern, 1985; Sroufe, 1995; Harrist and Waugh, 2002; Schore, 2005; Feldman, 2017; Hollenstein et al., 2017; Reindl et al., 2018). The specific socio-emotional task of the preschool years is to go from dyadic to self-regulation (Sroufe, 1995). In this period, synchronicity supports the acquisition of social skills (Harrist and Waugh, 2002) accompanied by the development of complex social emotions, such as shame and guilt, that signal the understanding of social standards and rules (Lewis, 2011; Botto and Rochat, 2018). The parental style that induces somaticity is intrusive defining-misattunement, which does not allow the child to reach proper security in self-definition (Guidano and Liotti, 1983; Guidano, 1987, 1991, 2007). As a result, the child tends to remain uncertain of their own internal states and in need for definition from a caregiver. Sensations and emotions – the most somatic states – are the first to be involved, but gradually the caregiver’s attitude extends to more abstract ones such as preferences and opinions. Typically, the caregiver adheres to conventional standards, and the child strives to comply with them. Somaticity can lead to an enmeshed family, where members have very undefined self-borders and tend to have common emotions and opinions (Minuchin et al., 1978).

##### Clinical

The caregiver intrusive defining-misattunement is causally related to the main clinical manifestations of somaticity – eating disorders (Guidano and Liotti, 1983; Guidano, 1987, 1991, 2007; Nardi et al., 2010). These disorders have been found (A) significantly correlated to attachment insecurity (Tasca and Balfour, 2014; Faber et al., 2018) and, in accordance with the somatic uncertainty about self-definition and tendency to compliance, (B) characterized by alexithymia (Schmidt et al., 1993; Westwood et al., 2017) and unassertiveness (Behar et al., 2006; Hartmann et al., 2010).

##### Evolutionary

In a difficult EEA, the child that gets imprinted to comply with the caregiver’s standards enhances their chances to survive by adopting a view of the situation that already proved functional instead of trying a new one. This becomes salient when the child develops the ability of autonomous exploration and starts to have broader social interactions (β-period, 2–6 years). The somatic adult will keep tending to comply with social standards, thereby improving their survival and reproductive chances in a harsh environment, where social compliance can make a key difference.

#### Obsessivity and the Judgmental Caregiver

We define obsessivity as the subjective measure of how much the caregiver is judgmental. The child needs to learn ethics – namely, what is considered right or wrong – from the caregiver, and the obsessive child experiences the imposition of the caregiver’s code of conduct and being significantly blamed for not abiding by it. In other words, the caregiver enforces a strict and arbitrary set of rules by systematically blaming the child for disobedience. The blame is always justified by claiming to cause terrible harm to someone and conveys the implicit message of the caregiver’s rejection of the child. It can assume different forms, such as verbal scold or physical punishment. For example, the child might be severely reproached for wearing wrinkled clothes (like in the case of Alison, Guidano, 1987). In this case, the child will tend to feel anguished by the possibility of misbehaving – even involuntarily – and to focus on acting correctly.

As a result, we propose, the preschool child gets imprinted an implicit knowledge that can be expressed by the core belief “I am wicked” (Table 3). This knowledge characterizes obsessivity making the obsessive particularly sensitive to ethical matters and inclined to suffer from (1) obsessions focused on their responsibility for causing harm and (2) compulsions to get rid of such intrusive ideas. In practice, obsessivity is revealed by such sensitivity.

Our definition and proposal are supported by the following evidence.

##### Caregiving-attachment

The moral guidance of the child is a fundamental caregiving task that has been widely studied, identifying three main related parenting styles: authoritative, authoritarian, and permissive (Baumrind, 1971, 2013; Robinson et al., 1995; Skinner et al., 2005). The acquisition of a code of conduct, accompanied by the emergence of guilt, is accomplished in the preschool years (Sroufe, 1995; Aksan and Kochanska, 2005; Lewis, 2011; Nicolais et al., 2017; Botto and Rochat, 2018) and has been found to have a significant impact on the next development of the child (Baumrind et al., 2010; Kochanska et al., 2010). The parental style that induces obsessivity is cold and severe authoritarianism (Guidano and Liotti, 1983; Guidano, 1987, 1991, 2007; Lennertz et al., 2010; Timpano et al., 2010). Typically, one of the two parents is very active in imposing the rules, and the other is a passive accomplice.

##### Clinical

The caregiver aloof and strict moral guidance is causally related to the main clinical manifestation of obsessivity – the obsessive-compulsive disorder (Guidano and Liotti, 1983; Guidano, 1987, 1991, 2007; Nardi et al., 2010). This disorder has been found (A) significantly correlated to attachment insecurity (Myhr et al., 2004; Doron et al., 2009; Ivarsson et al., 2010; Rezvan et al., 2012; Boysan and Çam, 2016) and, in accordance with the obsessive ethical focus, (B) characterized by guilt related to the violation of a moral rule (Shafran et al., 1996; Basile et al., 2013; Mancini and Gangemi, 2015).

##### Evolutionary

In a difficult EEA, the child that gets imprinted to respect the caregiver’s rules enhances their chances to survive by adopting a stricter and safer behavior. This becomes noticeable when the child develops locomotion and widens their social interactions, being both more cautious and a more reliable partner with a better reputation (β-period, 2–6 years). The obsessive adult, through conscientious application of their code, will reach relevant competencies (Hertler, 2015a,b), thereby improving their survival and reproductive chances in a harsh competitive environment.

#### Solution to the ‘Psychopathology Problem’ (P3)

The above arguments strongly suggest that: (A) The caregiver has four fundamental tasks: (1) regulating the child’s balance between attachment and exploration; (2) being emotionally reachable for the child; (3) regulating the child’s internal states and supporting the child’s own definition of them; (4) providing an ethical reference to the child. (B) These tasks become relevant during the preschool years. (C) The child needs to adapt to their caregiver’s particular way of accomplishing these tasks. (D) An optimal solution to this adaptation problem is to detect the caregiver’s attitude with respect to each of these tasks and rapidly and stably acquire such vital information – in other words, to imprint some core beliefs. Given their origin in the attachment relationship, these beliefs correspond to attachment dimensions and, given their fundamental socio-psychological role, they constitute the core of personality. (E) Particular kinds of accomplishments of these caregiving tasks induce the acquisition of core beliefs that can more easily lead to later dysfunctional states and behaviors – i.e., to psychopathology. (F) In particular, using the definitions that we propose: (1) A limiting caregiver induces phobicity, (2) an unreachable caregiver induces depressivity, (3) a defining caregiver induces somaticity, and (4) a judgmental caregiver induces obsessivity. Four caregiving features result to be causally related to four attachment dimensions.

Attachment theory could not find a specific connection between attachment and most psychopathologies because it investigated only the α-dimensions. In contrast, by considering the β-dimensions, the APM can indicate the specific causal relationship between attachment – and, therefore, personality – and the most common mental disorders (Table 5), thereby solving the problem (P1) of fully relating attachment to psychopathology.

### Imprinting and Sensitive Periods

As we discuss below, imprinting is an evolutionarily preordained unconscious learning process, whose main characteristics are: (1) Taking place for the first time in sensitive periods during the early stages of life; (2) Being particularly resistant to change. In the previous sections, we argued that attachment is characterized by seven dimensions and assumed that they are first acquired through imprinting over two early sensitive periods: (1) The α-period (6–24 months), in which the α-dimensions – disorganization, avoidance, and ambivalence – are imprinted; and (2) The β-period (2–6 years, the preschool age), in which the β-dimensions – phobicity, depressivity, somaticity, and obsessivity – are imprinted. In this section, we make a case for imprinting as the specific attachment acquisition mechanism by drawing on five different areas: (1) Ethological; (2) Developmental-Attachment; (3) Clinical; (4) Evolutionary; and (5) Neuroscientific.

#### Ethological Argument

Imprinting and sensitive periods have been documented in various species of birds and mammals (Lorenz, 1937, 1981; Harlow, 1959; Hess, 1959; Shipley, 1963; Salzen, 1967; Table 6).

**TABLE 6:** Sensitive periods in different species.

**Species**	**Approximate Sensitive Period**	**Imprinting**	**References**
Ducks	birth – 32 h	Identity	Hess, 1959
Geese	birth – 48 h	Identity	Lorenz, 1937; Troller-Renfree and Fox, 2017
Rats	birth – 10 days	Identity	Opendak and Sullivan, 2016; Opendak et al., 2017
Rhesus monkeys	2 weeks – 6 months	Identity	Harlow and Zimmermann, 1959; Harlow and Harlow, 1962; Harlow and Suomi, 1970; Suomi et al., 1974
Humans	2–6 months	Identity	Marvin et al., 2016; Troller-Renfree and Fox, 2017
	6 months – 2 years	α-Dimensions	
	2–6 years	β-Dimensions	Our proposal

*Sensitive periods and imprinting types in some species of birds and mammals are shown. Animals that are simpler in evolutionary terms – and more developed at birth – seem to show a more rudimental form of imprinting – with a sensitive period that is closer to birth and more clear-cut. We propose that humans, as the most complex beings on earth, have multiple attachment imprintings with overlapping sensitive periods. The imprinting of the caregiver identity is followed by that of the α- and β-dimensions.*

Goslings, for example, attach to their mother during a well-defined brief sensitive period shortly after hatching. Lorenz demonstrated that, when they catch some cues from the environment at the right time, they follow whoever matches the expected features. Famously, they attached to Lorenz himself. Through this – clearly evolutionarily preordained – process, the animal fixes in their mind the identity of their caregiver. Reasonably, given our psycho-social complexity, we-humans might have evolved a more elaborated version of imprinting compared to other animals. Indeed, we argue that we are not only preordained to fix in our mind the identity of specific caregivers, but also the most evolutionarily relevant attachment characteristics – the seven attachment dimensions.

#### Developmental and Attachment Argument

Bowlby (1969/1982) suggested the existence of imprinting and sensitive periods in humans noticing the striking similarities between our attachment and that of other animals, such as the geese studied by Lorenz (1937) or the rhesus monkeys studied by Harlow (1959). Since then, these concepts have been generally maintained by developmental-attachment research (Marvin et al., 2016), but the multiple intervening factors that can contribute to change seem to have caught much more attention than them. However, the α-period is unequivocally confirmed as sensitive for attachment by studies concerning early child-institutionalization that prove the long term effect of attachment experiences that occur within the first 2 years of life (Varin et al., 1996; Nelson et al., 2007; Zeanah et al., 2011; Fox, 2014; Troller-Renfree and Fox, 2017; Tables 1, 6). The effect of such experiences can be actually irreversible.

Regarding the β-period (preschool age), we can consider that:

1. Attachment develops beyond the second year of life (Bowlby, 1969/1982).
2. Although the child’s social context opens up to new interactions – in particular, with peers – (Nelson, 2007; Coplan and Arbeau, 2009; Marvin et al., 2016), the attachment relationship is still primary in the preschool years – since the child yet entirely depends on the caregiver for their survival (Crittenden, 2008; Marvin et al., 2016).
3. In the preschool years, caregiving is characterized by the four fundamental tasks provided by the caregiver’s β-features (cf. “Phobicity and the Limiting Caregiver,” “Depressivity and the Unreachable Caregiver,” Somaticity and the Defining Caregiver, and “Obsessivity and the Judgmental Caregiver”): (1) Protecting and supporting autonomy; (2) Being emotionally reachable; (3) Favoring self-definition; (4) Providing moral guidelines. These features represent four developmentally standard care-conditions to which the child needs to adapt.

All this is consistent with the imprinting of the four β-dimensions, which provides the child with vital information to adapt to the four human-specific attachment situations that become relevant during the β-period. As discussed above, we suggest that (Table 2):

1. The child adapts to an exploration-limiting caregiver by becoming phobic.
2. The child adapts to an emotionally unreachable caregiver by becoming depressive.
3. The child adapts to a state-defining caregiver by becoming somatic.
4. The child adapts to a judgmental caregiver by becoming obsessive.

#### Clinical Argument

Clinical research strongly supports the persistence of personality characteristics from childhood to adulthood and the correspondence between caregiving features and acquired attachment characteristics (Berne, 1972; Bowlby, 1973, 1980; Guidano and Liotti, 1983; Guidano, 1987, 1991; Blatt and Levy, 2003; Guidano, 2007; Levy et al., 2015; Yakeley, 2018). The resistance to change is evident in psychopathology. In cognitive psychotherapy, the phenomenon is called ‘neurotic paradox’ (Perdighe and Mancini, 2010), which refers to the apparently inexplicable persistence of mental disorders despite the patients being aware of their self-damaging behaviors. The phenomenon is consistent with dysfunctional knowledge that is imprinted and can be hardly modified. This is exactly the characteristic of the seven attachment dimensions and, in fact, all major psychopathologies can regularly be linked to information acquired in attachment relationships, as shown by clinical accounts that report early attachment information (e.g., Bowlby, 1973, 1980; Guidano and Liotti, 1983; Guidano, 1987, 1991; Oltmanns et al., 2012).

More specifically for the preschool age, as discussed above (cf. “Phobicity and the Limiting Caregiver,” “Depressivity and the Unreachable Caregiver,” Somaticity and the Defining Caregiver, and “Obsessivity and the Judgmental Caregiver”): (1) the β-dimensions are causally related to specific mental disorders; (2) The β-period is the earliest timeframe for the onset of such disorders; (3) These disorders tend to last throughout life; and (4) Parenting has been recognized as one of the major causes of their onset (Hopkins et al., 2013; Whalen et al., 2017). Since imprinting is the mechanism that underpins the long preservation of information, these data strongly suggest the imprinting of the β-dimensions in the preschool years.

#### Evolutionary Argument

Attachment has been designed by evolution as a fundamental adaptation mechanism. Bowlby (1969/1982; 1973; 1980) has first underlined the survival function of attachment for the child and, later, other authors (Chisholm, 1996; Chisholm and Sieff, 2014; Simpson and Belsky, 2016; Szepsenwol and Simpson, 2019; Young et al., 2019) have stressed the reproduction function of it for the adult. Indeed, the ultimate evolutionary goal of the individual is inclusive fitness (Hamilton, 1964), i.e., passing one’s gene to the following generations. These authors argue that the early attachment relationship provides cues of environmental characteristics – such as harshness and unpredictability (Ellis et al., 2009) – and the child unconsciously gathers these cues from the caregiver to set up an adequate strategy for later reproduction. This consideration suggests that attachment information acquired as a child is meant to be durable. Otherwise, it would lose its value for reproductive purposes. This is precisely what an imprinted IWM with its seven attachment dimensions is meant to provide.

In fact, this line of reasoning fits the preschool age. As discussed above (cf. “Phobicity and the Limiting Caregiver,” “Depressivity and the Unreachable Caregiver,” Somaticity and the Defining Caregiver,” and “Obsessivity and the Judgmental Caregiver”), beyond that in childhood, the β-dimensions have an adaptive function in adulthood: (1) Phobicity promotes protection through maintaining physical closeness; (2) Depressivity promotes the ability of being self-reliant; (3) Somaticity promotes familial cohesiveness through uniformity of thought and feeling; (4) Obsessivity promotes conscientiousness and competence through strict moral conduct. All these characteristics provide the adult with an advantage in a harsh and possibly unpredictable environment, thereby justifying imprinting the corresponding information from when it is available – the β-period indeed – to adulthood.

#### Neuroscientific Argument

The neural processes underlying attachment during sensitive periods have been identified and studied in different birds and non-human mammals (Knudsen, 2004; Sullivan and Holman, 2010; Landers and Sullivan, 2012; Knudsen, 2013; Nakamori et al., 2013; Roth et al., 2016; Feldman, 2017; Opendak et al., 2017; Opendak and Sullivan, 2019). These studies suggest the possible neural underpinnings of imprinting in the human brain. In particular, Knudsen (2004, 2013) describes how imprinting in sensitive periods corresponds to a shaping of neural networks that is durable but still admits of a later change through a particular interaction with the environment, which is exactly what is here hypothesized for the IWM. In accordance with Bowlby, it has also been established that, during a sensitive period, attachment to a caregiver occurs regardless of the care received, even when the caregiver is abusive (Sullivan and Holman, 2010; Landers and Sullivan, 2012; Opendak et al., 2017; Opendak and Sullivan, 2019), which further confirms the evolutionary programming of the process. Finally, lines of research across neuroscience and clinical psychology (Schore, 1994; Turnbull and Solms, 2003; Schore, 2009) identify the human brain areas involved in imprinting, with a sensitive period within the first 24 months (Tables 1, 6). In accordance with Bowlby, they stress the life-long durability of the phenomenon and its influence on personality.

Although neuroscience only recently has begun to address the attachment relationship directly in humans, some evidence has already been provided for a sensitive β-period. The studies of Rao et al. (2010) and Luby et al. (2016) found that maternal support in the preschool years is significantly correlated with hippocampal growth until adolescence, while maternal support in the following school years does not correlate with it. As a result, these authors explicitly propose that the preschool years are an attachment-related sensitive period. Moreover, Luby et al. (2016) found that preschool maternal support is correlated with emotional regulation in adolescence, with positive support linked to greater regulation ability. These data confirm the hypothesis of the preschool years as a sensitive period for attachment, with caregiving having a role in psychopathology. This is precisely what the APM proposes for the β-period and β-dimensions.

#### Solution to the ‘Stability Problem’ (P2)

The above five arguments converge to provide compelling support for imprinting as the specific attachment acquisition mechanism. The α-dimensions are imprinted in a sensitive α-period (6–24 months), and the β-dimensions in a sensitive β-period (2–6 years, the preschool age).

Therefore, the APM solves the dilemma of stability (P2) by clarifying that attachment – as it is expected of anything related to personality – is fundamentally stable.

## Discussion

Focusing on its information component, personality can be seen as the set of stable traits that are critically determined by one’s core beliefs. According to this perspective, to determine personality, we need to identify its core beliefs and their origin. Standard attachment theory offers a privileged starting point to solve this issue by connecting personality to the core knowledge that each of us acquires in early attachment relationships. However, the theory encounters three major problems concerning the link between attachment and personality: (P1) Intergenerational transmission; (P2) Stability, and (P3) Psychopathology.

In this article, by adequately enhancing the standard theory, we propose a solution to these problems that leads to the full account for the relationship between attachment and personality. We consider a total of seven attachment dimensions and the mechanism of their acquisition – imprinting. In particular, we suggest the following solutions: (P1) A specific caregiving feature realizes the intergenerational transmission of each attachment dimension (Table 2); (P2) Imprinting provides attachment with fundamental stability (Table 1); (P3) Psychopathology is causally related to specific attachment dimensions (Table 5).

These solutions clarify the general stability and fundamental socio-psychological value of the seven attachment dimensions, suggesting them to be the knowledge core of personality (Table 3).

### Implications

Overall, the APM entails significant consequences for attachment and personality theories and for the conception, assessment, and treatment of the most common mental disorders.

#### Implications for Attachment Theory

The APM proposes a revision of the current theory that is fully compatible with its achievements but, at the same time, can significantly enhance its explanatory power. In particular, the model suggests the relationship between caregiving features and attachment dimensions (P1), the basic stability of attachment due to imprinting (P2), and its connection to psychopathology (P3). We reach this result by focusing on the knowledge aspect of attachment and relying particularly on clinical research to explain the available data. This allows us to reduce complexity and identify key elements and their causal links. In contrast, standard attachment theory has been usually trying to take into account as many variables as possible relying mostly on statistical studies that cannot identify causal links. As a result, many variables have been considered, but the relationship between them has appeared often unclear.

In particular, we consider an IWM consisting of seven dimensions: 3 α-dimensions – disorganization, avoidance, and ambivalence – first imprinted in an α-period and four β-dimensions – phobicity, depressivity, somaticity, and obsessivity – first imprinted in a β-period (Table 1). We specify the definition of avoidance and ambivalence so that avoidance is transmitted via an emotional channel and ambivalence is transmitted via a physical channel. As a result, the α-dimensions are each induced by a specific caregiving feature. Furthermore, we introduce the four β-dimensions as each also induced by a specific caregiving feature. Consequently, we provide a complete mapping between caregiving and attachment (Table 2). According to the APM, the caregiving features correspond to fundamental tasks, among which the caregiver normally switches according to the situation, thereby inducing the acquisition or the expression of the corresponding attachment dimensions. On the other hand, given that they correspond to different caregiving features, the attachment dimensions are assumed to be independent, at least in terms of their function. However, since the same caregiver is usually responsible for the induction of multiple – if not all – dimensions, some characteristic patterns are to be expected. For example, when somaticity is the predominant dimension, then phobicity is also expected to be high because a defining caregiver will probably somehow limit their child’s exploration (Guidano, 1991). Finally, disorganization and the β-dimensions explain how attachment is linked to psychopathology (Table 5), as further discussed below.

We focus on dimensionality and imprinting as the central aspects of attachment that derive from its evolutionary predefinition. We propose that these seven dimensions specify the full range of adaptation-vital information, and imprinting provides the necessary stability to its acquisition – making it a durable and reliable adaptive base for the child and the future adult. In contrast, standard attachment theory has been exclusively concentrating on the information acquired within the first 2 years of life and on the possibility of its change. As a result, key aspects of attachment could not be identified.

#### Implications for Personality

Personality is a complex construct, and numerous models have been proposed from different perspectives. Between them, we focus here on one of the most influential and widely referred to: the Five-Factor Model (FFM) (or Big-Five) (McCrae and Costa, 2003). It consists of five traits – Openness, Conscientiousness, Extraversion, Agreeableness, Neuroticism – that have been statistically extracted from linguistic descriptions of personality characteristics. The model can be applied to healthy subjects but has also been linked to personality disorders (Widiger et al., 2017). An important feature of the FFM compared to the APM is its purely empirical nature with no endorsement of any specific etiological theory of personality. In other words, it is purely descriptive and cannot explain where the dimensions it postulates come from. In contrast, the APM is grounded in attachment theory – a theory of human relationships that finds in early experiences with the caregiver the foundation of personality, thereby offering an evident explanatory advantage. Moreover, in the condition of its applicability, we can expect the APM to have no less descriptive power than the FFM, since the set of α- and β-dimensions covers a broad range of personality features (Bowlby, 1969/1982, 1973, 1980; Guidano and Liotti, 1983; Guidano, 1987, 1991, 2007; Nardi et al., 2010; Liotti and Farina, 2011; Hesse, 2016; Mikulincer and Shaver, 2016). Interestingly, empirical studies have found correlation between the fundamental attachment dimensions of avoidance and ambivalence and the Big Five (Noftle and Shaver, 2006; Fraley et al., 2011). In this regard, the most relevant finding is the negative correlation of avoidance with agreeableness and the positive correlation of ambivalence with neuroticism. These results match the typical avoidant detachment and ambivalent over-emotionality. Given their meaning, we can also expect some correspondence between the β-dimensions and the FFM traits. Somaticity, for example, is expected to be correlated with agreeableness, obsessivity with conscientiousness.

The connection between the APM and the FFM needs to be further investigated and, at the moment, only preliminary hypotheses can be formulated about it. In this respect, a central issue concerns the possibility of personality change throughout life. The APM proposes personality to be resistant to change, but empirical findings underpinned by the FFM indicate that changes are to be expected (Lucas and Donnellan, 2011; Oltmanns et al., 2019). For example, agreeableness and conscientiousness have been found to increase between age 20 and 60, while openness to decrease after 60, with important individual differences (Roberts and Mroczek, 2008). A ‘maturity principle’ has been proposed (Caspi et al., 2005; Lucas and Donnellan, 2011) according to which agreeableness and conscientiousness tend to increase and neuroticism to decrease over young and middle adulthood in relation to the assumption of mature social roles. The end of maturity (Oltmanns et al., 2019) has also been identified, corresponding to the decrease of extraversion, conscientiousness, and openness from late middle age. In particular, personality stability and change over the life course have been connected to identity processes (Roberts and Caspi, 2003). According to this perspective, continuity is related to identity strength, in terms of achievement and certainty, for example. Changes of identity that are characteristic of different stages of life – and related to different social roles (e.g., being independent, having a family, pursuing a career) – would explain corresponding changes of personality (Roberts et al., 2003, 2006). Although the apparent contrast, we can hypothesize that these findings are fully compatible with the general stability endorsed by the APM since the APM and the FFM focus on different constructs. First, the two models seem to measure different social variables related to personality. The APM is based on attachment, which is a social phenomenon limited to close relationships, while the above studies suggest that FFM measures are sensitive to broader social aspects. Second, the APM concerns the knowledge part of personality, whereas the high heritability of the FFM traits (Jang et al., 1996; Power and Pluess, 2015) suggests them to be more sensitive to biological variables and related changes over the lifespan. The indispensable empirical testing of the APM will provide further data to elaborate on this point.

#### Implications for Psychopathology

The implications of the APM for psychopathology are even stronger than those for personality.

##### Attachment disorders

As discussed above, the β-dimensions express core beliefs (Table 3) that clearly correspond to the cognitive organizations (CO) first identified by Guidano and Liotti (Table 4), who demonstrated the causal connection of the COs to the most common mental disorders. As a result, these dimensions are causally connected to specific psychopathologies (Table 5). The APM proposes the attachment origin of the β-dimensions, thereby connecting the vast and valuable clinical research related to the COs to a wider attachment framework. This link entails remarkable consequences. The two immediate ones are the following:

A. Any disorder that can be traced back to a β-dimension has an attachment etiology. These disorders are usually the most common ones – such as agoraphobia, depression, anorexia, bulimia, obsessive–compulsive disorder. Clearly, when a disorder has a β-dimension at its root, attachment plays a major role in its unfolding.
B. Attachment is also the principal mechanism underlying healing from these conditions. Since the change of a dimension is bound to attachment activation, the healing process implies the action of an attachment figure. In other words, when therapy is successful, the therapist offers through their – usually implicit – caregiving what has been called a ‘corrective emotional experience’ (Alexander and French, 1946; Mallinckrodt, 2010). The APM specifies the features of this experience – the successful intervention must have involved the patient’s pathological dimensions. Of course, the intervention can be tailored to each patient by addressing their specific pathological dimensions.

Therefore, the APM suggests that – in these cases – the attachment relationship is both (A) the key pathogenic factor and (B) the key healing one. Of course, the same considerations apply to disorganization and related dissociative disorders. Consequently, the model provides the basis for an etiological classification of psychopathology, with clear significant impact on assessment and treatment.

##### Pathological dynamics and treatment

The APM can be linked to the general motivational dynamics as described by Liotti’s motivational theory (Liotti and Monticelli, 2008; Liotti et al., 2017), which integrates attachment in a more general motivational framework. According to this view, human behavior can be described as underpinned by the continuous activation of built-in motivational systems, which drive us to pursue evolutionarily relevant goals – such as exploration, sex, attachment, caregiving, cooperation. If a pathology is related to attachment, the activation of the attachment motivational system will be potentially problematic. The APM suggests that five specific domains – corresponding to disorganization and the β-dimensions – can cause issues when attachment is activated: a situation will more easily elicit a pathological reaction if it concerns a dysfunctional dimension. For example, a threat will be more problematic if one is disorganized, a separation if one is phobic, a loss if one is depressive. Given that a pathological attachment dimension is connected to intolerable internal states (Bowlby, 1969/1982, 1973, 1980; Guidano and Liotti, 1983; Guidano, 1987, 1991; Schore, 1994; Sroufe, 1995; Schore, 2000; Liotti and Farina, 2011; Brumariu, 2015), the individual who has such an issue will develop – from the preschool years – some strategies to avoid the activation of attachment in relation to the given dimension. These can be seen as strategies to regulate attachment activation – activating a non-attachment system to avoid that of attachment. For example, a child could activate caregiving with their mother, thereby taking care of her and inverting the attachment relationship (Bowlby, 1973). Regulation strategies have been identified for disorganization (Hennighausen and Lyons Ruth, 2005; Lyons-Ruth and Jacobvitz, 2008; Liotti, 2011; Liotti and Farina, 2011) and ambivalence and avoidance (Shaver and Mikulincer, 2002; Mikulincer et al., 2003; Mikulincer and Shaver, 2016). The APM suggests that they extend to the β-dimensions. This view is very well supported. Indeed, the dysfunctional attachment patterns described by Crittenden in the preschool years (Crittenden, 1995; 2008; Farnfield et al., 2010) are evident regulation strategies. Moreover, providing the attachment foundation to the COs, the APM can be immediately integrated with the clinical approaches stemmed from Guidano and Liotti’s research (Guidano, 2007; Nardi and Bellantuono, 2008; Arciero and Bondolfi, 2009; Liotti and Monticelli, 2014; Liotti et al., 2017). Finally, the concepts of attachment-related core belief and regulation strategy are analogous to those of interpersonal schema and interpersonal cycle (Safran and Segal, 1996; Dimaggio, 2015). As a consequence, as mentioned above, the APM is also perfectly compatible with the well-established metacognitive interpersonal therapy (Carcione et al., 2016; Dimaggio et al., 2017, 2019; Gordon-King et al., 2019). Therefore, the APM – in accordance also with the TAM model – suggests the attachment origin of our central psycho-social beliefs and is consistent with the fundamental clinical role of metacognition.

In conclusion, we can stress that, given the close relationship between psychopathology and attachment, mental disorders can offer us important insights into the nature of personality. Since one’s core beliefs are related to common psychopathologies, the study of such conditions can shed light on the structure and possible change of personality.

### Limitations and Suggestions for Future Work

We suggest here the two main limitations of our model and possible ways to overcome them.

#### Scope

The APM refers to the aspects of personality related to imprinted attachment data. Therefore, when other variables – such as the biological ones – have a non-negligible influence on personality, such variables should be taken into account. We think that complexity should be added gradually to the model, by considering new variables – and upgrading the model itself – but only after adequate testing.

#### Testing

Although we found significant support for the hypotheses we formulate here, the APM is a novel theoretical model. As a consequence, it will need to be thoroughly empirically validated, especially through the operationalization and measurement of each dimension. In particular, we can consider the main hypotheses formulated by the APM – (H1) The existence of seven dimensions of attachment, each with specific characteristics; (H2) The correspondence between these dimensions and specific caregiving features; (H3) The role of the dimensions in personality and psychopathology – and suggest three different strategies (that we are already implementing) to test them.

##### Clinical

The most natural testing for the APM appears to be the clinical one, which can be performed not only directly, in clinical practice, but also indirectly, through the examination of case studies that include sufficient information about the attachment history of the patient (e.g., Bowlby, 1973, 1980; Guidano and Liotti, 1983; Guidano, 1987, 1991; Oltmanns et al., 2012). All the above main hypotheses are testable by analyzing clinical data, but especially H3 for its relevance in dysfunctional conditions. So far, this testing has fully confirmed the model. A systematic review of a larger number of cases is an optimal strategy for further testing.

##### Statistical

Questionnaires are a well-established assessment practice in attachment theory (Barone and Del Corno, 2006). The proposed dimensionality of attachment (H1) and correspondence between caregiving features and attachment dimensions (H2) (Table 2) can be empirically tested through a questionnaire administered to adults who answer about their current and past experiences.

##### Computational

Attachment relationships can be represented mathematically and modeled computationally (Buono et al., 2006; Amengual, 2009; Stevens and Zhang, 2009; Petters and Beaudoin, 2017) – for example, by representing mother and child in a software virtual environment. A mathematical model that is built in accordance with a given theory allows for the testing of its hypotheses. If the simulations do not reproduce the data gathered in real situations, the corresponding assumptions are disconfirmed. Since the APM integrates attachment into the whole motivational dynamics and offers a dimensional view of attachment knowledge, it is particularly suitable for computational implementation. However, given the complexity of pathological circumstances, this kind of testing seems to be more adequate to test H1 and H2 rather than H3. Interestingly, the simulation environment can work as a lab of synthetic psychology (Braitenberg, 1984; Dawson, 2004; Prescott and Camilleri, 2018), where not only attachment phenomena can be reproduced, but new situations can be generated to further test and extend the model.

## Conclusion

We fully agree with those psychologists who believe that theoretical investigation is as important as the empirical one to the development of Psychology (Borghi and Fini, 2019). In this spirit, we present here a novel theory and hypotheses, urging their direct empirical testing. This work represents our attempt to contribute to the theoretical framework of clinical psychology and its connections to other fields of psychology and connected disciplines. Over the years, a great amount of evidence pointing to the fundamental role of attachment in the building of personality has been accumulating. The APM gives a precise form to this idea: During two consecutive sensitive periods early in life, humans acquire from their caregivers essential cues through which they set their fundamental socio-psychological variables corresponding to seven attachment dimensions. Seven core beliefs are imprinted to be the knowledge foundation of our personality, which will give us implicit directions throughout our lives. We are predefined adaptive machines designed by evolution to be unconsciously programmed by our caregivers.

## Data Availability Statement

The original contributions presented in the study are included in the article/supplementary material, further inquiries can be directed to the corresponding author/s.

## Author Contributions

The author confirms being the sole contributor of this work and has approved it for publication.

## Conflict of Interest

The author declares that the research was conducted in the absence of any commercial or financial relationships that could be construed as a potential conflict of interest.
